# Supplementary material for: Phenotypic and Molecular Study of Multidrug-Resistant Escherichia coli Isolates Expressing Diverse Resistance and Virulence Genes from Broilers in Tunisia
Source: Antibiotics (Basel). 2025 Sep 15;14(9):931. doi: 10.3390/antibiotics14090931 (PMC12466712; doi:10.3390/antibiotics14090931)
Supplement: Supplementary file 1 [file antibiotics-14-00931-s001.zip › antibiotics-3799029-supplementary.pdf]

Supplementary files:

Table S1. Primers used in the present study for the detection antimicrobial resistance and integrons genes of *E. coli* isolated from ceca of healthy poultry.

Assay	Target gene	Primers sequences (5′-3′)	Annealing temperature (°C)	Size of PCR product (bp)	PCR conditions	Reference
β-lactamase genes (ESBL)	$bla_{CTX-M-grp\ 1}$	F: ATGGTTAAAAAATCACTGCG<br>R: TTACAAACCGTCGGTGAC	49°C	876		[78]
	bla_{SHV}	F: CACTCAAGGATGTATTGTG<br>R: TTAGCGTTGCCAGTGCTCG	54°C	885		[79]
	ba_{TEM}	F: ATTCTTGAAGACGAAAGGGC<br>R: ACGCTCAGTGGAACGAAAAC	50°C	1150		[79]
Cephalosporinase	bla_{CMY}	F: ATGATGAAAAAATCGATATG<br>R: TTATTGCAGTTTTTCAAGAATG	55°C	1146		[80]
Carbapenemase	bla_{OXA-48}	F: GCGTGGTTAAGGATGAACAC<br>R: CATCAAGTTCAACCCAACCG		438	Quadruplex-PCR	[81]
	bla_{NDM-1}	F: GGTTTGGCGATCTGGTTTTC<br>R: CGGAATGGCTCATCACGATC	55°C	621		
	bla_{KPC}	F: CGTCTAGTTCTGCTGTCTTG<br>R: CTTGTCATCCTTGTTAGGCG	55°C	798		
	bla_{IMP}	F: GGAATAGAGTGGCTTAAYTCTC<br>R: GGTTTAAYAAAACAACCACC	55°C	203		
	bla_{VIM}	F: GATGGTGTTTGGTCGCATA<br>R: CGAATGCGCAGCACCAG	52°C	390		[21]
Colistin	*mcr-1*	F: CGGTCAGTCCGTTTGTTC<br>R: CTTGGTCGGTCTGTAGGG	54°C	309		[82]
Quinolones	*qnrA*	F: AGAGGATTTCTCACGCCAGG<br>R: TGCCAGGCACAGATCTTGAC	57°C	580	Triplex PCR	[83]
	qnrB	F: GCMATHGAAATTCGCCACTG<br>R: TTTGCYGYYCGCCAGTCGAA		264		
	qnrS	F: GCAAGTTCATTGAACAGHGGT<br>R: TCTAAACCGTCGAGTTCGGCGCG		428		
	aac(6)′-Ib	F: TTGCGATGCTCTATGAGTGGCTA<br>R: CTCGAATGCCTGGCGTGTTT	53°C	482		[84]
Aminoglycoside	*aac(3)-II*	F: ACTGTGATGGGATACGCGTC<br>R: CTCCGTCAGCGTTTCAGCTA	57°C	300		[85]
	aadA(1-2)	F: GCAGCGCAATGACATTCTTG<br>R: ATCCTTCGGCGCGATTTTG	54°C	300		[77]
	aadA(5)	F: CTTCAGTTCGGTGAGTGGC<br>R: CAATCGTTGCTTTGGCATAT	53°C	500		[77]
Sulfamide	*Sul1*	F: TGGTGACGGTGTTCGGCATTC<br>R: GCGAGGGTTTCCGAGAAGGTG	62°C			[77]
	Sul2	F: CGGCATCGTCAACATAACC	50° C			[77]

		R: GTGTGCGGATGAAGTGAG				
	Sul3	F: CATTCTAGAAAACAGTCGTAGTTCG<br>R: CATCTGCAGCTAACCTAGGGCTTTGGA	51°C			[77]
Integrons	*IntI1*	F: GGGTCAAGGATCTGGATTTCG<br>R: ACATGCGTGTAAATCATCGTCG	62°C	483 bp		[77]
	IntI2	F: CACGGATATGCGACAAAAAGGT<br>R: GTAGCAAACGAGTGACGAAATG	62°C	788bp		[77]
	qacEΔ1 + *sul1*	F: GGCTGGCTTTTTCTTGTTATCG<br>R: GCGAGGGTTTCCGAGAAGGTG	63°C	1125 bp		[77]
	Variable region of the integron class 1	F: GGCATCCAAGCAGCAAG<br>R: AAGCAGACTTGACCTGA	55°C	Variable		[77]
	Variable region of the integron class 1	F: GATGCCATCGCAAGTACGAG<br>R: CGGGATCCCGGACGGCATGCACGA	60°C	variable		[77]

Table S2. Primers used in this study for the detection virulence genes and phylogenetic grouping of *E. coli* isolated from ceca of healthy poultry.

PCR reaction	Gene	Primer sequence (5′-3′)	Size of PCR product (bp)	Annealing temperature (°C)	PCR conditions	Refer
	**Phylogenetic genes**					
Quadruplex	*chuA*	chuA.1b: ATGGTACCGGACGAACCAAC chuA.2: TGCCGCCAGTACCAAAGACA	288	60	Quadruplex-PCR	[7]
	yjaA	yjaA.1b: CAAACGTGAAGTGTCAGGAG yjaA.2b: AATGCGTTCCTCAACCTGTG	211	60		[7]
	TspE4C2	TspE4C2.1b: CACTATTCGTAAGGTCATCC TspE4C2.2b: AGTTTATCGCTGCGGGTCGC	152	60		[7]
	arpA	AceK.f: AACGCTATTCGCCAGCTTGC AceK.r: TCTCCCCATACCGTACGCTA	400	60		[7]
Group E	*arpA*	ArpAgpE.f: GATTCCATCTTGTCAAAATATGCC ArpAgpE.r: GAAAAGAAAAAGAATTCCCAAGAG	301	57		[7]
Group C	*trpA*	trpAgpC.1: AGTTTTATGCCCAGTGCGAG trpAgpC.2: TCTGCGCCGGTCACGCCC	219	59	*Duplex-PCR*	[7]
Internal control	*trpA*	trpBA.f: CGGCGATAAAGACATCTTCAC trpBA.r: GCAACGCGGCCTGGCGGAAG	489	57		[7]
**Virulence factors**						
Shiga toxin type 1	*stx1*	F: CAGTTAATGTGGTGGCGAAGG R: CACCAGACAATGTAACCGCTG	348 bp	56	Quadruplex-PCR	[86]
Shiga toxin type 2	*stx2*	F: ATCCTATTCCCGGGAGTTTACG R: GCGTCATCGTATACACAGGAGC	584 bp	56		[86]
Enterohaemolysin	*ehxA*	F: GCATCATCAAGCGTACGTTCC R: AATGAGCCAAGCTGGTTAAGCT	534 bp	56		[86]

Enteropathogenic attachment and effacement	*Eae*	F : TGCGGCACAACAGGCGGCGA R : CGGTCGCCGCACCAGGATTC	629 pb	56		[86]
type 1 fimbriae	*FimH*	F: TGCAGAACGGATAAGCCGTGG R: GCAGTCACCTGCCCTCCGGTA	506	56°C	Quadruplex-PCR	[87]
serum survival gene	*traT*	F:GGTGTGGTGCGATGAGCACAG R: CACGGTTCAGCCATCCCTGAG	290	56°C		[87]
invasion of brain endothelium	*ibeA*	F: AGGCAGGTGTGCGCCGCGTAC R: TGGTGCTCCGGCAAACCATGC	170	56°C		[87]
S and F1C fimbriae	*Sfa/foc*	F: CTCCGGAGAACTGGGTGCATCTTAC R: CGGAGGAGTAATTACAAACCTGGCA	410	56°C		[87]
cytolethal distending toxin	*cdt3*	F: GAAAATAAATGGAATATAAATGTCCG F: TTTGTGTCGGTGCAGCAGGGAAAA	555	56°C	Quadruplex-PCR	[88]
hemolysin	*Hly*	F: GAGCGAGCTAAGCAGCTTG R: CCTGCTCCAGAATAAACCACA	889	56°C		[89]
cytotoxic necrotizing factor	*Cnf1*	F: GGGGGAAGTACAGAAGAATTA F: TTGCCGTCCACTCTCTCACCAGT	1111	56°C		[88]
malate dehydrogenase (Mdh)	*Mdh*	F: AGGCGCTTGCACTACTGTTA R: AGCGCGTTCTGTTCAAAT G	835 bp	56°C		[90]
aerobactin system	*iutA*	F: GGCTGGACATCATGGGAACTGG R: CGTCGGGAACGGGTAGAATCG	300	63°C	*Duplex-PCR*	[87]
yersiniabactin	*fyuA*	F: TGATTAACCCCGCGACGGGAA	880	63°C		[87]

		R: CGCAGTAGGCACGATGTTGTA				
papG allele III (P *fimbriae* adhesin	*papGIII*	F: CATTTATCGTCCTCCTCAACTTAG<br>R: AAGAAGGGATTTTGTAGCGTC	482	55°C		[91]
P fimbriae	*papA*	F: ATGGCAGTGGTGTCTTTTGGTG<br>R: CGTCCCACCATACGTGCTCTTC	717	56°C		[87]
aerobactin system	*Aer*	F: TACCGGATTGTCATATGCAGACCGT<br>R: AATATCTTCCTCCAGTCCGGAGAAG-	602	63°C		[92]
bundle forming pilus	*bfpA*	F: AATGGTGCTTGCTTGCGGCTTGCTGC<br>R: GCCGCTTTTATCCAACCTGGTA	323 bp	56°C		[93]

Table S3. Characteristics of the 111 *E. coli* isolates were obtained from caeca of chicken broilers samples.

Code souche	ESBL	Phylog-roups	Virulence genes	B-lactamase genes	Non-B-lactamase genes	*Int1*	*qacEΔ1 + sul1*	VR1*	*Int2*	VR2*
ME1-X	+	A	*traT, fimH, ibeA, iutA*	$bla_{CTX-M-G1}$, bla_{TEM}	*aadA-1, sul1*	+	-		-	
MK1	+	C	*traT, fimH, iutA*	bla_{SHV}, bla_{TEM}	*qnrB, aac(6)Ib-cr, aadA-1, sul1*	+	+	*dfrA1-aadA1*	-	
ME4X	+	Clade I	*traT, fimH, BfpA, iutA, fyuA*	$bla_{CTX-M-G1}$, bla_{TEM}		+	+		+	
ME5X	+	B1	*traT, fimH, BfpA, iutA*	$bla_{CTX-M-G1}$, bla_{SHV}, bla_{TEM}	*acc(3)-II, aadA-1*	+	-		+	
MK6	+	A		$bla_{CTX-M-G1}$, bla_{SHV}	*acc(3)-II*	+	-		-	
ME6X	+	A	*traT, fimH, BfpA, iutA*	$bla_{CTX-M-G1}$	*aadA-5*	+	-		+	
ME7	+	B1	*traT, fimH*	$bla_{CTX-M-G1}$	*qnrB, sul1*	+	+		-	

ME7X	+	A	*traT, fimH, BfpA, iutA*	$bla_{CTX-M-G1}$		+	-		-	
ME8X	+	B1	*traT, fimH, BfpA, iutA*	$bla_{CTX-M-G1}$, bla_{SHV}	*aadA-1*	+	-		-	
ME9X	+	D	*traT, fimH, iutA, fyuA*	bla_{SHV}, bla_{TEM}, bla_{CMY}	*aadA-1, sul1*	+	+		-	
ME10X	+	F	*traT, iutA*	$bla_{CTX-M-G1}$, bla_{TEM}, bla_{CMY}	*aadA-1, sul1*	+	+		+	*sat2-dfrA1*
ME11X	+	B1	*stx-1, traT, fimH, BfpA*	$bla_{CTX-M-G1}$, bla_{SHV}, bla_{TEM}		-	-		+	
ME12X	+	A		$bla_{CTX-M-G1}$, bla_{SHV}, bla_{TEM}	*acc(3)-II, aadA-1*	-	+		-	
ME13X	-	E	*traT, fimH, iutA*	$bla_{CTX-M-G1}$, bla_{SHV}, bla_{TEM}	*acc(3)-II, aadA-1*	-	-		+	
ME14X	-	F	*traT, fimH, iutA*	$bla_{CTX-M-G1}$, bla_{SHV}, bla_{TEM}	*acc(3)-II, aadA-1, aadA-5*	-	-		-	
ME15X	+	C	*traT, fimH, BfpA, iutA, fyuA*	$bla_{CTX-M-G1}$, bla_{SHV}, bla_{TEM}	*aadA-1*	+	-		-	
ME16X	+	E	*traT, fimH, BfpA, iutA*	$bla_{CTX-M-G1}$, bla_{TEM}		+	-		-	
ME17X	-	E	*traT, fimH, iutA, fyuA*	$bla_{CTX-M-G1}$, bla_{SHV}, bla_{TEM}		-	+		+	
ME18X	+	D	*stx-1, fimH*	$bla_{CTX-M-G1}$	*aac(6)Ib-cr, acc(3)-II, aadA-1*	+	+	*dfrA12-aadA2*	-	
ME19X	+	E	*traT, BfpA, iutA*	$bla_{CTX-M-G1}$, bla_{TEM}		+	-		-	
ME20X	-	E	*traT, fimH, iutA*	$bla_{CTX-M-G1}$, bla_{TEM}	*acc(3)-II, aadA-1*	-			+	
ME21X	-	D	*stx-1, traT, fimH, iutA*	$bla_{CTX-M-G1}$, bla_{SHV}, bla_{OXA-48}	*acc(3)-II*	+	-		-	
ME22X	+	A	*eae, traT,fimH, iutA*	$bla_{CTX-M-G1}$, bla_{TEM}, bla_{IMP}	*aac(6)Ib-cr, aadA-1*	+	+		-	
ME23X	+	A	*eae, traT, fimH*	$bla_{CTX-M-G1}$	*aadA-1*	+	-		-	
ME24X	-	D	*traT, fimH, BfpA, iutA, fyuA*							

ME25X	-	F	*traT, fimH, iutA, fyuA*	*bla*CTX-M-G1	*aadA-1*	+	+		+	*Sat2-aadA1*
ME26X	-	D	*traT, fimH, iutA, fyuA*							
ME27X	-	F	*traT, fimH, iutA, fyuA*	*bla*CMY						
ME28X	-	D	*traT, fimH, iutA, fyuA*							
ME29X	+	A	*eae, traT, fimH*	*bla*CTX-M-G1		+	-		-	
ME30X	+	A	*eae, traT, fimH*	*bla*CTX-M-G1		+	-		-	
ME31X	-	F	*traT, fimH, iutA, fyuA*	*bla*SHV, *bla*TEM, *bla*CMY	*qnrB, aadA-1*	+	+		+	
ME32X	-	F	*traT, fimH, iutA, fyuA*		*qnrB, aadA-1*	+	-		+	*Sat2-aadA1*
ME33X	-	F	*traT, fimH, iutA, fyuA*	*bla*CTX-M-G1, *bla*TEM, *bla*CMY	*aadA-1*	+	+		+	*sat2-dfrA1*
ME34X	+	B1	*stx-1, sfa/focDE, BfpA*	*bla*TEM	*aadA-1*	+	-		+	
ME35X	-	F	*stx-1, traT, fimH, iutA, fyuA*	*bla*SHV, *bla*TEM	*aadA-1*	+	-		+	*sat2-dfrA1*
ME36X	+	A	*stx-1, eae, traT, fimH*	*bla*CTX-M-G1, *bla*SHV		+	-		+	
ME37X	-	F	*traT, fimH, iutA*	*bla*CTX-M-G1, *bla*SHV		-	-		+	
ME38X	+	B1	*traT, fimH, iutA*	*bla*CTX-M-G1, *bla*TEM	*qnrB, aadA-1*	+	-		+	
ME39X	-	F	*stx-1, eae, iutA*	*bla*CTX-M-G1, *bla*SHV, *bla*TEM, *bla*CMY		+	-		+	*sat2-dfrA1*
ME40X	+	A	*traT, fimH, iutA*	*bla*CTX-M-G1, *bla*SHV	*aadA-1*	+	-		+	
ME41X	+	B1	*traT, fimH, BfpA, iutA, fyuA*	*bla*CTX-M-G1, *bla*SHV	*aadA-1*	+	-		+	
ME42X	+	B2	*stx-1, fimH*	*bla*CTX-M-G1, *bla*SHV		+	-		+	

ME43X	+	F	*traT, fimH, BfpA, iutA, fyuA*	bla_{SHV}, bla_{TEM}	*aadA-5*	+	-		+	*Sat2-aadA1*
ME44X	+	B2	*stx-1, traT, fimH, iutA, fyuA*	$bla_{CTX-M-G1}$	*aadA-5*	+	-		-	
ME45X	+	Clade I	*stx-1, stx-2, traT, fimH, iutA*	$bla_{CTX-M-G1}$, bla_{TEM}	*aadA-1*	+	-		+	
ME46X	+	A	*traT, BfpA, iutA*	bla_{SHV}, bla_{TEM}	*aadA-1*	+	-		+	
ME47X	+	A	*stx-2, BfpA, iutA*	$bla_{CTX-M-G1}$, bla_{SHV}, bla_{TEM}	*acc(3)-II, aadA-1*	-	-		+	
ME48X	+	B1	*stx-1, eae, cdt3, fimH, BfpA, fyuA*	$bla_{CTX-M-G1}$, bla_{IMP}		-			-	
ME49X	-	F	*traT, fimH, BfpA, iutA, fyuA*	$bla_{CTX-M-G1}$, bla_{TEM}	*aadA-1*	+	-		+	*sat2-dfrA1*
ME50X	+	B2	*stx-1, eae, traT, fimH, iutA, fyuA*	$bla_{CTX-M-G1}$, bla_{TEM}	*acc(3)-II, aadA-1*	+	-		-	
ME51X	+	D	*stx-1, traT, fimH, iutA, papGIII*	$bla_{CTX-M-G1}$, bla_{SHV}	*acc(3)-II, aadA-1*	+	-		+	
ME52X	+	D	*stx-1, traT, fimH, BfpA, iutA, fyuA*	bla_{SHV}, bla_{CMY}, bla_{OXA-48}	*acc(3)-II, aadA-1, aadA-5*	+	+		-	
ME53X	+	B1	*stx-1, iutA*	bla_{TEM} bla_{CMY}, bla_{OXA-48}	*mcr-1, aadA-1*	+	-	*dfrA1-aadA1*	-	
ME54X	-	B1	*stx-1, traT, fimH, papGIII*	$bla_{CTX-M-G1}$, bla_{CMY}	*mcr-1*	+	+	*dfrA1-aadA1*	-	
ME55X	+	F	*stx-1, traT, fimH, iutA, fyuA*	$bla_{CTX-M-G1}$, bla_{OXA-48}	*aadA-1, aadA-5*	+	-		-	
ME56X	+	F	*stx-1, traT, fimH, BfpA, iutA*	$bla_{CTX-M-G1}$, bla_{OXA-48}	*aac(6)Ib-cr, aadA-1, aadA-5*	+	-		-	

ME57 X	+	F	*stx-1, traT, fimH, BfpA, iutA, fyuA, papGIII*	*bla*$_{CTX-M-G1}$, *bla*$_{SHV}$	*aac(6)Ib-cr, aadA-5*	+	-		-	
ME58 X	+	A	*traT, fimH, BfpA, iutA*	*bla*$_{CTX-M-G1}$, *bla*$_{SHV}$	*qnrB, qnrS, aac(6)Ib-cr, aadA-1, sul1*	+	-		-	
ME59 X	+	D	*stx-1, traT, fimH, BfpA, iutA, fyuA*	*bla*$_{CTX-M-G1}$, *bla*$_{SHV}$, *bla*$_{OXA-48}$		+	-		-	
ME60 X	+	A	*traT, fimH, iutA*	*bla*$_{CTX-M-G1}$, *bla*$_{TEM}$	*qnrB, qnrS, aadA-1, aadA-5, sul1*	+	-		-	
ME61 X	+	A	*traT, fimH, BfpA*	*bla*$_{CTX-M-G1}$	*qnrB, aadA-1, aadA-5*	+	-		-	
ME62 X	+	A	*traT, fimH, BfpA*	*bla*$_{CTX-M-G1}$, *bla*$_{SHV}$	*qnrB, qnrS, aac(6)Ib-cr, aadA-1, aadA-5, sul2*	+	-		-	
ME63 X	+	A	*stx-1, stx-2, traT, fimH, BfpA, iutA*	*bla*$_{SHV}$, *bla*$_{CMY}$	*qnrS, Aac(6)Ib-cr, aadA-1, aadA-5*	+	+		-	
ME64 X	+	B1	*stx-1, stx-2, traT, fimH, BfpA, iutA*		*qnrB, aadA-1, aadA-5, sul1, sul2*	+	+	*aadA1*	-	
ME65 X	+	B1	*stx-1, traT, fimH, BfpA, iutA*	*bla*$_{SHV}$	*qnrB, acc(3)-II, aadA-1, sul1, sul2*	+	+	*dfrA1-aadA1*	-	
ME66 X	-	B1	*ehxA, traT, fimH, BfpA, iutA*	*bla*$_{TEM}$, bla$_{CMY}$	*qnrA, aac(6)Ib-cr, acc(3)-II, sul2*	+	-		-	
ME67 X	+	D	*traT, fimH, BfpA, iutA*	*bla*$_{CTX-M-G1}$, *bla*$_{SHV}$, *bla*$_{TEM}$	*qnrB, qnrS, aac(6)Ib-cr, acc(3)-II, aadA-1, aadA-5, sul1, sul2*	+	+	*dfrA1-aadA1*	-	
ME68 X	+	D	*traT, fimH, BfpA, iutA,*	*bla*$_{CTX-M-G1}$, *bla*$_{TEM}$	*qnrB, qnrS, aac(6)Ib-cr, acc(3)-II, aadA-1, aadA-5, sul1, sul2*	+	+	*dfrA1-aadA1*	-	
ME69 X	+	A	*traT, fimH, iutA*	*bla*$_{CTX-M-G1}$	*mcr-1, qnrA, qnrS, aac(6)Ib-cr, aadA-1*	+	+		-	
ME70 X	+	A	*stx-1, traT, fimH, BfpA, iutA*	*bla*$_{CTX-M-G1}$	*qnrS, aac(6)Ib-cr, aadA-1, aadA-5*	+	-		-	

ME71 X	+	B2	*stx-1, ehxA, cdt3, traT, fimH, BfpA, iutA, fyuA*		*aac(6)Ib-cr, aadA-1, sul2*	+	-		+	*dfrA 1-Sat2*
ME72 X	+	A	*stx-1, traT, fimH, BfpA, iutA, fyuA*	*bla*$_{CTX-M-G1}$, *bla*$_{IMP}$	*qnrS, aac(6)Ib-cr, aadA-1, aadA-5*	+	-		+	*dfrA 1-Sat2*
ME73 X	+	A	*stx-1, cdt3, traT, fimH, BfpA, iutA*	*bla*$_{CTX-M-G1}$, *bla*$_{IMP}$	*aac(6)Ib-cr, aadA-1, aadA-5*	+	-		-	
ME74 X	+	B1	*stx-1, fimH, sfa/focDE, BfpA, iutA*	*bla*$_{SHV}$	*qnrS, aac(6)Ib-cr, aadA-1, aadA-5, sul2*	+	-		+	
ME75 X	+	A	*stx-1, fimH, sfa/focDE, BfpA, iutA, fyuA, papGIII*	*bla*$_{CTX-M-G1}$, *bla*$_{SHV}$	*aadA-1, aadA-5*	+	+		+	
ME76 X	+	B1	*stx-1, traT, fimH, BfpA, iutA, fyuA*		*aac(6)Ib-cr, aadA-1, aadA-5, sul1, sul2*	+	+	*dfrA1-aadA1*	+	
ME77 X	+	B1	*stx-1, traT, fimH, BfpA, iutA, fyuA*	bla$_{OXA-48}$	*aadA-1, aadA-5, sul1*	+	+	*dfrA1-aadA1*	+	
ME78 X	+	D	*stx-1, fimH*			-			-	
ME79 X	+	D	*stx-1, traT, fimH*	*bla*$_{CTX-M-G1}$, *bla*$_{TEM}$	*qnrS, aac(6)Ib-cr, aadA-1, aadA-5, sul1, sul2*	+	+	*aadA1*	+	
ME80 X	-	A	*traT, fimH*	*bla*$_{CTX-M-G1}$	*mcr-1, qnrS, aac(6)Ib-cr, aadA-1, aadA-5, sul1, sul2*	+	+		+	
ME81 X	+	B2	*stx-1, fimH, BfpA, iutA, papGIII*	*bla*$_{CTX-M-G1}$	*qnrB, aac(6)Ib-cr, aadA-1, aadA-5*	+	-		+	
ME82 X	-	A	*stx-1, eae, traT, fimH, BfpA,*	*bla*$_{CTX-M-G1}$, *bla*$_{TEM}$	*qnrB, qnrS, acc(3)-II, aadA-1, aadA-5, sul1, sul2*	+	+	*dfrA12-aadA2*	+	

			iutA, fyuA							
ME83X	-	A	*traT, fimH, BfpA, iutA*	*bla*$_{CTX-M-G1}$, *bla*$_{TEM}$ *bla*$_{OXA-48}$	*qnrS, aac(6)Ib-cr, aadA-1, aadA-5*	-			-	
ME84X	-	D	*fimH, iutA*	*bla*$_{CTX-M-G1}$, *bla*$_{OXA-48}$	*qnrS, aac(6)Ib-cr, aadA-1, sul2*	+			-	
ME85X	-	A	*stx-1, traT, fimH, iutA*	*bla*$_{CTX-M-G1}$	*mcr-1*	-			-	
ME86X	-	C	*traT, fimH, BfpA, iutA, papGIII*	*bla*$_{CTX-M-G1}$, *bla*$_{TEM}$, *bla*$_{OXA-48}$	*qnrS, aac(6)Ib-cr, aadA-1*	+			-	
ME87X	-	B2	*stx-1, stx-2, eae, traT, iutA*	*bla*$_{CTX-M-G1}$, *bla*$_{TEM}$	*qnrB, aac(6)Ib-cr, acc(3)-II, sul2*	+	-	*aadA1*	+	
ME88X	-	A	*traT*	*bla*$_{CTX-M-G1}$, *bla*$_{OXA-48}$	*aac(6)Ib-cr, aadA-1*	-			-	
ME89X	+	A	*traT, iutA, fyuA*	*bla*$_{CTX-M-G1}$, *bla*$_{OXA-48}$	*aac(6)Ib-cr, acc(3)-II, aadA-1*	+	+		-	
ME90X	-	A	*traT*	*bla*$_{CTX-M-G1}$, *bla*$_{OXA-48}$	*qnrB, aac(6)Ib-cr, aadA-1*	+	+		-	
ME91X	-	A	*stx-1, traT, fimH, BfpA*		*qnrB, aac(6)Ib-cr, acc(3)-II, aadA-1, sul2*	+	+		+	
ME92X	-	A	*stx-1, stx-2, traT, fimH, iutA, fyuA*	*bla*$_{CTX-M-G1}$	*qnrB, aac(6)Ib-cr, acc(3)-II, sul2*	+	-		+	
ME93X	+	B1	*traT, fimH, iutA, fyuA*	*bla*$_{CTX-M-G1}$	*acc(3)-II, aadA-1*	+	+		+	*dfrA1-Sat2*
ME94X	+	B1	*stx-1, eae, BfpA*		*aadA-1*	+	+		-	
ME95X	+	E	*stx-1, ehxA, traT, fimH, iutA*		*aadA-1*	+	-		-	
ME96X	+	A	*traT, fimH, BfpA, iutA, papGIII*	*bla*$_{CTX-M-G1}$ *bla*$_{TEM}$, *bla*$_{IMP}$	*qnrS, aac(6)Ib-cr, aadA-1, aadA-5, sul2*	+	+		-	
ME97X	+	A	*traT, fimH, BfpA, iutA*	*bla*$_{CTX-M-G1}$, *bla*$_{SHV}$, *bla*$_{TEM}$, *bla*$_{CMY}$	*mcr-1, qnrS, aac(6)Ib-cr, aadA-1, aadA-5, sul2*	+	+		+	*dfrA1-Sat2*
ME98X	+	F	*traT, iutA*	*bla*$_{CTX-M-G1}$	*aadA-5*	+	+		-	
ME99X	-	A	*traT, iutA*	*bla*$_{CTX-M-G1}$, *bla*$_{TEM}$, *bla*$_{IMP}$	*qnrS, aadA-5*	-			-	

ME100X	+	E		$bla_{CTX-M-G1}$	*aadA-1*	-			-	
ME101X	+	E	*traT, fimH*	$bla_{CTX-M-G1}$, bla_{TEM}, bla_{OXA-48}	*aadA-1*	+	+		-	
ME102X	+	D	*traT, fimH, BfpA*	$bla_{CTX-M-G1}$, bla_{IMP}	*qnrB, acc(3)-II, aadA-1, aadA-5*	+			-	
ME103X	+	A	*traT, fimH, BfpA, fyuA*	$bla_{CTX-M-G1}$, bla_{TEM}	*qnrS, acc(3)-II, aadA-1, sul1, sul2*	+	+		-	
ME104X	+	A	*stx-1, iutA*	$bla_{CTX-M-G1}$	*aadA-1, aadA-5*	+	+		+	
ME105X	+	A	*fimH, fyuA*	$bla_{CTX-M-G1}$, bla_{TEM}	*qnrB, acc(3)-II, aadA-1*	+	+		-	
ME106X	+	A	*traT, fimH, iutA*	$bla_{CTX-M-G1}$, bla_{TEM}	*qnrS, acc(3)-II, aadA-1*	+		*dfrA12 - aadA2*	-	
ME107X	+	unknown	*traT*	$bla_{CTX-M-G1}$	*aadA-1*	+	+		-	
ME108X		A	*stx-2, eae, traT,fimH*	$bla_{CTX-M-G1}$	*acc(3)-II, aadA-1*	+	+	*dfrA12 - aadA2*	+	
ME109X	+	A	*fimH, iutA*	$bla_{CTX-M-G1}$, bla_{TEM}	*qnrS, aadA-1, sul2*	+	+		-	
ME110X	+	A	*stx-1, stx-2, eae, traT, fimH*	$bla_{CTX-M-G1}$	*qnrB, acc(3)-II, aadA-1, sul1*	+	+	*dfrA12 - aadA2*	-	
ME111X	+	A	*traT, fimH, iutA, fyuA*	$bla_{CTX-M-G1}$, bla_{TEM}	*qnrS, acc(3)-II, aadA-1, sul1, sul2*	+	+		-	

*VR1; variable region of class 1 integron, VR2; variable region of class 2 integron.
